# Quantifying Spatial Disparities in Neonatal Mortality Using a Structured Additive Regression Model

**DOI:** 10.1371/journal.pone.0011180

**Published:** 2010-06-17

**Authors:** Lawrence N. Kazembe, Placid M. G. Mpeketula

**Affiliations:** 1 Applied Statistics and Epidemiology Research Unit, Mathematical Sciences Department, Chancellor College, University of Malawi, Zomba, Malawi; 2 Biology Department, Chancellor College, University of Malawi, Zomba, Malawi; Aga Khan University, Pakistan

## Abstract

**Background:**

Neonatal mortality contributes a large proportion towards early childhood mortality in developing countries, with considerable geographical variation at small areas within countries.

**Methods:**

A geo-additive logistic regression model is proposed for quantifying small-scale geographical variation in neonatal mortality, and to estimate risk factors of neonatal mortality. Random effects are introduced to capture spatial correlation and heterogeneity. The spatial correlation can be modelled using the Markov random fields (MRF) when data is aggregated, while the two dimensional P-splines apply when exact locations are available, whereas the unstructured spatial effects are assigned an independent Gaussian prior. Socio-economic and bio-demographic factors which may affect the risk of neonatal mortality are simultaneously estimated as fixed effects and as nonlinear effects for continuous covariates. The smooth effects of continuous covariates are modelled by second-order random walk priors. Modelling and inference use the empirical Bayesian approach via penalized likelihood technique. The methodology is applied to analyse the likelihood of neonatal deaths, using data from the 2000 Malawi demographic and health survey. The spatial effects are quantified through MRF and two dimensional P-splines priors.

**Results:**

Findings indicate that both fixed and spatial effects are associated with neonatal mortality.

**Conclusions:**

Our study, therefore, suggests that the challenge to reduce neonatal mortality goes beyond addressing individual factors, but also require to understanding unmeasured covariates for potential effective interventions.

## Introduction

Despite declining trends in childhood mortality in many developing countries [1], neonatal mortality still remains a huge health concern worldwide [2], [3],[4]. Recent estimates from national-wide household surveys show that considerable burden of neonatal mortality still remain in low to middle-income countries, the majority of which are in the sub-Saharan Africa [2], [3]. Experts now agree that in evaluating Millennium Development Goal (MDG) number 4, which emphasizes for the need to reduce under-five childhood and infant mortality [5], neonatal mortality is a key child survival indicators to monitor. It is argued that achieving a reduction in neonatal mortality would also lead to a reduction in infant mortality [4].

The underlying causes of neonatal mortality are multi-sectoral and inter-woven [6]. These operate at individual, family, community and regional levels and the effects can be direct or intermediary. At individual level, the relationship between socio-economic and bio-demographic factors and neonatal mortality are well established [1], [7], [8]. Most of these factors act directly. At family level the intermediary factors are the shared genetic factors, sanitation and inadequate health care factors [6], [9]. Availability of antenatal and prenatal care as well as differences in ethnic norms and practices are some of the factors influencing disparities in child mortality at community level. Regionally, expenditure on health services and cultural differences can also affect the survival status of children in the neonatal period.

Evidently, the combined effect of all these factors are likely to cause geographical disparities in childhood mortality, even so, in neonatal mortality. Studying the geographical variation of neonatal mortality is of particular interest because access to antenatal or reproductive care vary and there exist regional differences in availability of services [10], hence newborn health may vary. Findings from such a study could assist in the design of effective interventions.

Analysis of spatially indexed data is common in biomedical and epidemiological research, in recognisance of the effect of geographical location on health outcomes. There is now an increasing body of literature on spatial analysis of health system and outcomes in developing countries [11], [12], [13], [14], [15], [16]. In part, this has been motivated by the availability of geo-referenced survey data, and further, by the recent advances in software that can implement such complex models [17]. Such analysis is carried out under the assumption that not all factors of the underlying process can be measured, and therefore a source of heterogeneity. These residual heterogeneity are in part likely to exhibit spatial dependence. The common approach to analyse such spatially referenced data is to incorporate, in the model, random effects that allow latent area influences.

**Figure 1:**
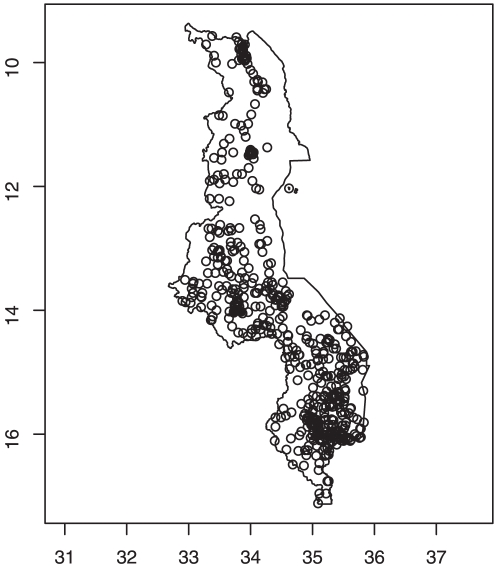
Survey data location. Neonatal mortality data: Locations where survey data was collected based on 2000 Malawi Demographic and Health Survey.

**Figure 2:**
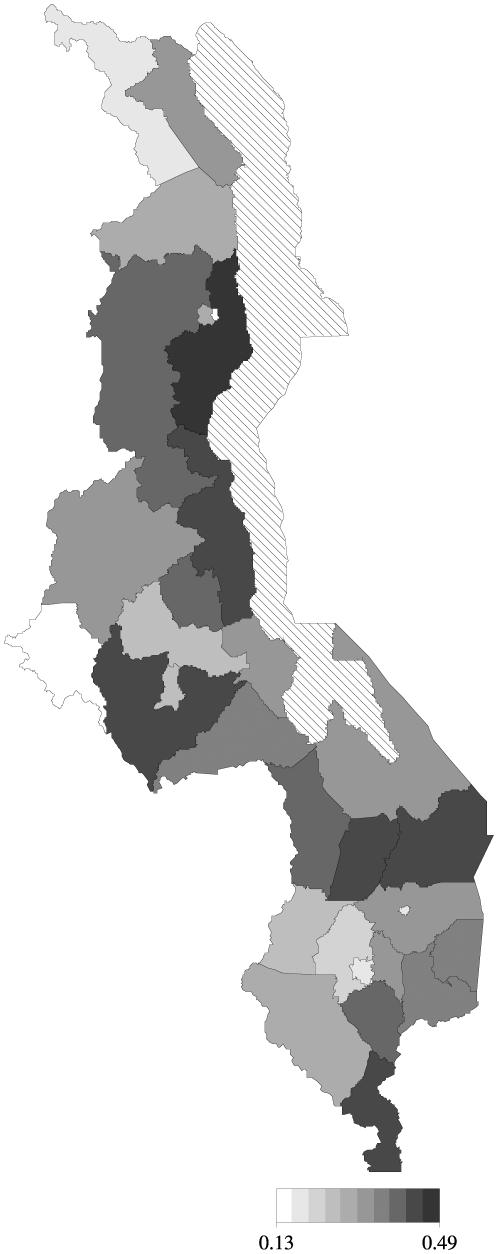
Estimated district proportion dead. Estimated district proportion died under the independent fixed-effects model.

In this paper, our objective is to analyze small-scale geographical variability in neonatal mortality in Malawi, by applying existing spatial statistical methodology [18], [19]. Since the outcome consists of a success (1 = if death occurs in the first four months) or failure (0 = otherwise), a Bernoulli model comes initially to mind; and in the absence of strong prior information, the first choice is a fixed-effects binary logistic model. However, the presence of geo-referenced data allows us to explore, assess and quantify small-scale geographical effects in neonatal mortality. Figure 1 shows the residential locations of the cases obtained in 2000 Malawi demographic and health survey (MDHS). Apparent clustering is due to the survey design [20]. The same information can be grouped at district level, and shown as proportion of neonatals dead in each area (Figure 2). Our aim is to extend the standard binary logit model to random-effects model to permit spatial clustering and heterogeneity. Specifically, we apply generalised linear mixed models (GLMM) with spatially correlated random effects proposed by [19], and used it to analyse factors associated with the survival status of infants during the first four weeks of life. This modelling approach falls within what is termed structured additive regression (STAR) models, introduced by [21]. STAR models are a comprehensive class of models that permit simultaneous estimation of nonlinear effects of continuous covariates, spatially unstructured and structured components together with the usual fixed effects in the predictor [19], [22], [23].

When the place of residence is known exactly, given by geographical *x*−*y*-coordinates, the spatial analysis can be approached based on the stationary Gaussian random fields (GRF), originating from geostatistics [18], [24]. These can also be interpreted as two-dimensional surface smoothers based on radial basis functions, and have been employed by Kammann and Wand (2003) to model the spatial component in Gaussian regression models. Another option is to use two-dimensional P-splines described in more detail in Lang and Brezger (2004) and Brezger and Lang (2006). The advantage of these approaches is that they allows prediction of risk for locations where there are no data, thus able to quantify small-scale variability. If observations are clustered in geographical regions, spatial effects can be estimated using the Markov random field (MRF) approach, widely used in disease mapping [24]. Modelling and inference can use the empirical Bayesian (EB) approach via penalised likelihood techniques [25]. However, fully Bayesian (FB) approach is possible [23].

The rest of this paper is structured as follows. Section 2 describes the data, while Section 3 gives details of the methodology used. In Section 4, we provide simulation studies and apply the techniques to real data from 2000 Malawi DHS. Section 5 gives the results and offers a discussion of the analysis. The final section is the conclusion.

## Materials and Methods

### 2.1 Data

The data were from the 2000 Malawi DHS [20]. The 2000 Malawi DHS interviewed a representative sample of more than 13,000 women aged between 15 and 49 years. A two-stage stratified sampling design was implemented to collect the data. The data were realized through a questionnaire that included questions on marriage and reproductive histories, of which detailed dates of birth of all women and their children were collected. Details on how the sample survey was designed, implemented, response errors and sampling errors are given in the survey report [20].

Women were asked histories of all births they ever had. Survival time of each child was then computed in months. All children whose survival time was less than 1 month were classified as neonatal deaths. The response, 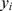, was therefore binary which takes the value 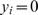 if infant *i* survived the first four week and 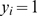 if the infant died. Covariates considered were bio-demographic variables including birth multiplicity (i.e. singleton or multiple birth), the sex of the child, birth interval preceding or succeeding the child in question, birth size, birth order and prenatal care indicators. Socio-economic variables included in the analysis were mother's education, area of residence (urban/rural) and care situation and practices of the mother. All the above were modelled as categorical variables. Further, continuous covariates considered were mother's age, and woman status. Women's status is defined to be women's power relative to men. The index about women's status is built following suggestions by [26]. For spatial covariates, we used both the exact geo-coordinates of enumeration areas and subdistricts as geographical units of analysis.

Descriptive summaries of the variables are reported in Table 1. Figure 1 shows the distribution of the MDHS study locations. There were 543 points, with mean number of households selected for interview per enumeration area equal to 36 (range: 6–68). Urban areas and other districts were over-sampled for correct population estimates, hence more data points in some areas than others. Complete data was available for 11,926 of the 13,220 interviewed. A total of 1559 children died within 5 years preceding the survey. Of these 543 (34.5%) died in the first 4 weeks of their life (neonatal period). Figure 2 gives estimates of the proportion of infants who died in each district, using a fixed-independent district model.

**Table 1:** Descriptive summary of factors analysed in neonatal mortality study in Malawi (2000 DHS).

Variables		Proportion died	No of births
*Socio-economic factors:*			
Region	Northern	4.0	1936
	Central	4.4	4394
	Southern	4.8	5596
Residence	Urban	2.8	2084
	Rural	4.9	9842
Mother's education	None	4.0	3547
	Primary	5.0	7513
	Secondary or higher	3.1	886
Antenatal Visits	None	8.8	297
	Once	3.1	3100
	Twice	2.9	2876
	Three or more	2.6	1668
Place of birth	Home	5.4	5047
	Hospital	4.0	6879
Woman's Status	Lowest	4.7	2618
	Low	4.0	2389
	Medium	4.4	2399
	High	4.9	2589
	Highest	4.8	1932
*Bio-demographic factors*			
Sex of child	Male	5.1	5951
	Female	4.0	5975
Multiplicity of birth	Singleton	3.9	11432
	Multiple	20.2	494
Birth order	1^st^	6.4	2883
	2–3	4.2	4707
	4–6	3.5	3263
	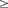 7 births	4.5	1573
Mother's age	<20 yrs	8.4	885
	20–24	5.0	3704
	25–29	4.1	3302
	30–34	2.5	1816
	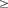 35 yrs	4.5	2219

### 2.2 Statistical Modelling

#### 2.2.1 The measurement model

We describe the spatial pattern of neonatal mortality given locations by adapting the hierarchical Bayesian model formulation of [19]. Let the response, 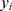, be the survival status of child *i* at location 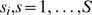. Define 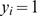 if the infant died within the first 4 weeks of life and 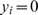 otherwise, then 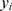 is a Bernoulli variable with expected probability of dying equal to 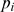. This can be modelled through the logistic regression model, i.e.,
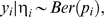
(1)

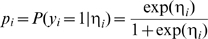
(2)where 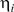 is the predictor. The predictor can be expanded as follows, taking into account all possible explanatory variables,
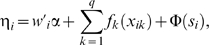
(3)such that α is the vector of fixed effects (e.g. sex of child, mother's education) corresponding to the categorical fixed variables 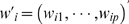, the component *f* is an appropriate smoothing function of continuous covariate, 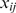, such as age of the mother. The parameter 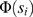 are random effects that captures the unobserved spatial heterogeneity at location 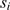. Some of these may be spatially structured and others spatially unstructured, which may accommodate over-dispersion and heterogeneity. In other words, 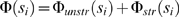. Accommodating these structures, equation 3 can be extended as
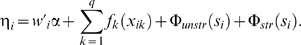
(4)This equation specifies the first stage of the hierarchical model. Written in matrix notation, equation (4) is given as
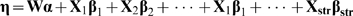
(5)which reduces to **η = Pθ**, where 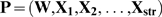 are appropriate design matrices for each fixed, metrical and spatial effects respectively, and 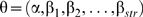 is a high dimensional parameter vector. The elements 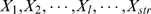 and 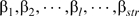 are such that 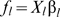, and for the spatial component, we can write as 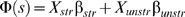.

#### 2.2.2 Prior distributions

In order to model the relationship depicted in equation 5, we specified prior distributions for each parameter in the model (eq. 5). Essentially this is the second stage of the hierarchy. For the fixed regression parameters, α, a suitable choice is the diffuse prior, i.e *p*(α)∝*constant*. The smooth functions of continuous covariates are modelled using a second-order random walk prior given by 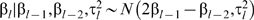 for *l* = 3,…,*b* with noninformative priors for 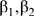. Again 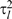 controls the amount of smoothing, with larger values leading to less smoothing. In order to capture unstructured spatial random effects (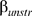), we assumed exchangeable normal priors, 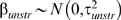, where 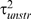 is a variance component that allows for over-dispersion and heterogeneity. Often determination of potential nonlinearity and spatial heterogeneity is chosen a priori based on exploratory analysis.

Spatial correlation between areas is achieved by incorporating suitable spatial correlation into 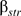. This is specified using either the MRF or GRF priors. The MRF is defined as
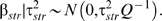
(6)where 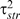 is the unknown precision parameter which controls the degree of similarity, and *Q* is the spatial precision matrix. The (*i*,*j*)-th element of the spatial precision matrix *Q* is given by
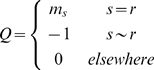
where *s*∼*r* denotes that area *s* is adjacent to *r*, 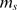 is the number of adjacent areas to *s*. We define areas as neighbours if they share a common border. Thus area *s*, given neighbouring areas *r*, has the following conditional distribution
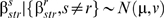
(7)where 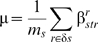, 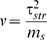 and *s* and *r* are adjacent areas in the set of all adjacent areas (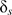) of area *s*, and 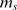 are the number of adjacent areas. For the variance components 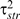 we assume inverse Gamma priors *IG*(*a*,*b*), with hyperparameters *a* = 0.01, *b* = 0.01.

Another option for spatial analysis, if exact locations 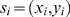 are available, is to use two-dimensional P-splines. To fit a spatial surface structure, the approach one can adopt is based on a two-dimensional P-spline suggested in [27]. A similar approach on thin plates has been recently proposed by [28]. We assume that the unknown surface 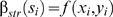 can be approximated by the tensor product of the one-dimensional P-splines, i.e.
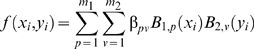
(8)where 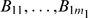 are the basis functions in 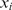 direction and 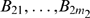 in 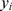 direction. The design matrix 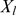 is now *n*×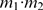 dimensional and consists of products of basis functions. Priors for 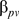 are based on spatial smoothness priors as specified in [29]. A two-dimensional first order random walk has been shown to work well [27]. This is based on the four nearest neighbours and is specified as
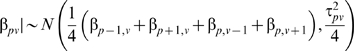
(9)for *p*,*v* = 2,…,*m*−1 and appropriate changes for corners and edges. This prior is a direct generalization of a first order random walk in one dimension. Its conditional mean can be interpreted as a least squares locally linear fit at knot position 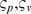 given the neighbouring parameters. In many applications it is desirable to additionally incorporate the 1 dimensional main effects. Again, similar to the one dimensional case additional identifiability constraints have to be imposed on the functions.

Using the design matrix 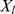 and a (possibly high-dimensional) vector of regression parameters 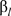, as defined above (see following eq. 5), the spatial and nonlinear smoothing priors can be expressed in a general Gaussian form
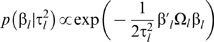
(10)with an appropriate penalty matrix 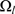. Its structure depends on the covariate and smoothness of the function. In most cases, 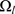 is rank deficient and hence the prior for 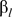 is improper.

#### 2.2.3 Empirical Bayesian approach

Inference for the semiparametric binary model is based on the empirical Bayesian approach, also called the mixed model methodology [17], [19]. The EB approach is achieved by recasting the predictor model (5) as GLMM after appropriate reparametrization. This provides the key for simultaneous estimation of the functions 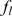 and the variance parameters 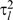 in the empirical Bayes approach. To rewrite model (5) as mixed model, we assume that 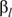 has dimension 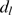 and the corresponding penalty matrix has rank 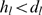 = dim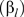. Each parameter vector 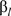 is partitioned into a penalized (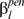) and unpenalized (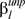) parts yielding a variance component model [17], [19],
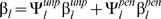
(11)for some well defined 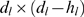 matrix 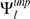 and a 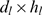 matrix 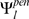. The following priors are assumed. For the penalized part, an i.i.d Gaussian prior is suitable, while for the unpenalized part we assume a flat prior, this is
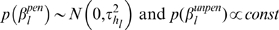
(12)Applying decomposition (11) to all the components of predictor (5) yields
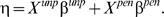
(13)We have obtained in (13) a GLMM with fixed effects 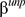 and random effects 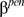. The posterior, in terms of the GLMM representation, is given by
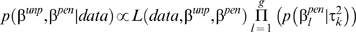
(14)where *L*(·), again, denotes the likelihood which is the product of individual likelihood contributions and 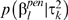 as defined above. Estimation of regression coefficients and variance parameters is carried out using iteratively weighted least squares and approximate restricted maximum likelihood. Details are given in [30]. Fahrmeir et al. [19] derived numerically efficient formulae that allow for handling large data sets.

### 2.3 Analysis

The empirical Bayes model described in Sections above is illustrated by analysing the small-scale spatial variability in neonatal mortality in Malawi using data from the 2000 Demographic and Health Survey. We fit the following five STAR models to assess factors associated with probability of neonatal mortality,

M0: 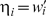


M1: 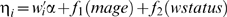


M2: 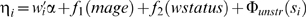


M3: 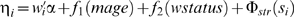


M4: 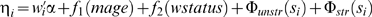.

The first model, which we denote as the baseline model (M0) estimated fixed effects, while second model (M1) adds the nonlinear terms of mother's age 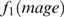 and women's status 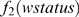, with no random effects. The third model, M2, extended model M1, and included the unstructured spatial random effects 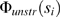. The fourth model M3 considered spatially structured random effects 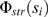, added to the fixed effects model (M0). Finally, the fifth model M4, included both structured and unstructured spatial effects, besides the fixed effects and the nonlinear terms. For the structured spatial effect we assume a first-order intrinsic Gaussian MRF prior (7) and two-dimensional P-spline prior (9). The GRF approach will not be considered since similar results are expected. On the spatial unit of analysis, using the MRF prior, we fit district and subdistrict in separate models because of limited structural variability. However, a multilevel aspect to the data in that subdistricts (TA/Ward) are nested within 31 districts may be relevant, but has not been fitted here. Such models are considered elsewhere [14].

The EB implementations of the five STAR models were implemented in BayesX - version 1.4 [17]. In the EB approach, estimation follows two stages. At the first iteration the default (starting) values are assumed for the penalized, unpenalized and variance parameters. Then updates for 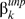 and 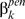 are obtained in the first step by solving a system of linear equations given estimates for the variance parameters. In the second step updates of the variance parameters are obtained by maximizing the approximate restricted log-likelihood. For each model fitted, convergence is achieved when the change in regression parameters is 0.0001 and terminated at 400 iterations if convergence is not achieved. However at under 35 iterations all models converged.

Model selection, among a set of competing models of various specifications, was based on Akaike information criterion (AIC), although generalized cross validation (GCV) or Bayesian information criterion (BIC) give similar conclusions. For a model with *df* degrees of freedom, AIC is defined as AIC(*df*) = −2×(max log-likelihood) + 2 *df*. The log-likelihood comprises the collection of all fixed effects, α, random effects β, and all random effects variances, 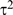. Smaller value of AIC or BIC signified a better model, that is models with Δ*AIC*<2 compared to the best model are to be considered as equally similar to the best model, whereas models with Δ*AIC*>4 can be weakly differentiated, and Δ*AIC*>10 indicate virtually no support.

## Results

Based on the AIC, model M0 has AIC = 4373.15, while model M1 gave an AIC of 4042.55, suggesting that the combined effect of individual characteristics and unstructured random effects explained the risk of neonatal mortality better than fixed effects alone. Now, incorporating the structured effects to the individual effects improved the model further (AIC = 4011.68 for model M3 versus AIC = 4373.15 in model M0). In the last model, the fit slightly improved when both structured and unstructured spatial effects were included in model (AIC for model M4 was 4009.58). Results for model M0, M3 and M4 are given in Table 2. However we discuss the best model (M4).

**Table 2:** Posterior estimates in the geoadditive logistic regression models M0, M3 and M4 fitted to neonatal mortality.

Variable	Category	1Model 0	2Model 3	3Model 4
Birth size	Smaller	0	0	0
	Average and above	−0.193 (−0.241, −0.149)	−0.202 (−0.250, −0.151)	−0.201 (−0.249, −0.154)
Sex of child	Girl	0	0	0
	Boy	0.065 (0.023, 0.108)	0.069 (0.027, 0.114)	0.068 (0.027, 0.111)
Multiple birth	Yes	0	0	0
	Singleton	−0.460 (−0.527, −0.391)	−0.465 (−0.537, −0.389)	−0.468 (−0.535, −0.394)
Birth order	1st	0.197 (0.082, 0.318)	0.204 (0.089, 0.318)	0.205 (0.089, 0.325)
	2–3	0.024 (−0.066, 0.114)	0.025 (−0.065, 0.116)	0.026 (−0.068, 0.116)
	4–6	−0.084 (−0.178, 0.011)	−0.088 (−0.184, 0.004)	−0.090 (−0.180, 0.003)
	7th and higher	0	0	0
Antenatal visits	None	0	0	0
	Once	−0.172 (−0.256, −0.091)	−0.179 (−0.267, −0.094)	−0.181 (−0.268, −0.097)
	Twice	−0.179 (−0.271, −0.083)	−0.186 (−0.277, −0.096)	−0.184 (−0.276, −0.095)
	3 or more	−0.165 (−0.282, −0.051)	−0.162 (−0.289, −0.049)	−0.164 (−0.278, −0.062)
Birth place	Home	0	0	0
	Hospital	−0.037 (−0.082, 0.002)	−0.041 (−0.086, 0.002)	−0.042 (−0.087, 0.003)
Residence	Urban	0	0	0
	Rural	0.091 (0.022, 0.159)	0.098 (0.025, 0.173)	0.095 (0.022, 0.166)
Mother's education	None	0	0	0
	Primary	0.115 (0.043, 0.193)	0.117 (0.036, 0.204)	0.123 (0.050, 0.201)
	Secondary or above	−0.108 (−0.245, 0.017)	−0.099 (−0.246, 0.038)	−0.097 (−0.238, 0.035)
−2×log-likelihood:		4335.39	3769.70	3763.54
Degrees of freedom:		18.98	120.99	122.98
AIC:		4373.15	4011.68	4009.58

1 Model 0: Fixed effects.

2 Model 3: Fixed+Nonlinear effects+Structured random effects.

3 Model 4: Fixed+Nonlinear effects+Structured+Unstructured random effects.

We first discuss the linear effects shown in Table 2. Results indicate that infants with birth weight above average (>2500 grams), born as singletons, born of mothers who sought antenatal care and those whose mother's attained secondary or higher education were all associated with lower probability of dying in the neonatal period. The effect of being a boy child, first born, born in rural area, and born to a mother who attained primary education was positively associated with neonatal deaths. Many of these effects are as expected, and are well known and studied [1], [4], [9], [31], [32].

**Figure 3:**
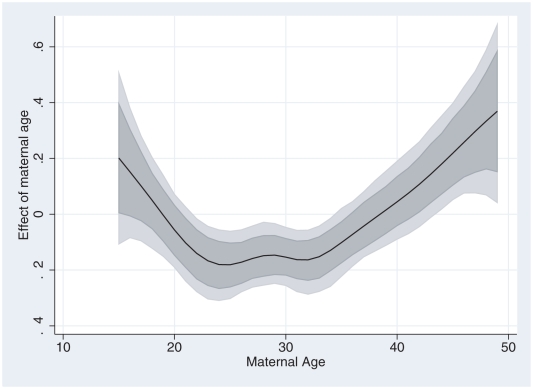
Nonlinear effect of mother's age. Nonlinear effect of mother's age on the risk of neonatal mortality (solid centre line), with 80% and 95% confidence lines (dotted lines).

**Figure 4:**
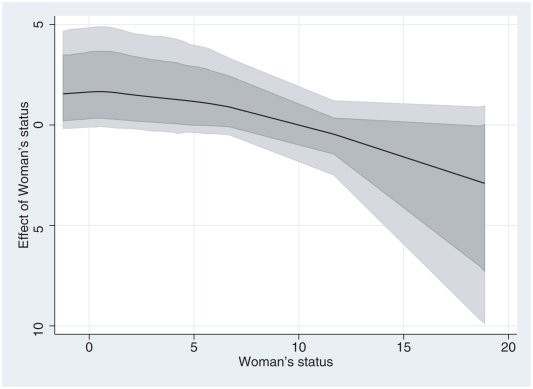
Nonlinear effect of mother's status. Nonlinear effect of mothers status on the probability of neonatal mortality (solid centre line), with 80% and 95% confidence lines (dotted lines).

The nonlinear effects are shown in Figure 3 and 4. Figure 3 shows posterior model estimates of mother's age together with 80% and 95% pointwise confidence intervals. There was a strong nonlinear effect, depicted as U-shape, of mother's age on the probability of neonatal mortality. The risk decreased as mother's age increased from 15 years up to 25 years, and then started to increase again after age 35 years. This behaviour is not unexpected. Lower maternal age increases the risk of pre-term birth, hence increased neonatal deaths. At old age deteriorating maternal health increases the risk of neonatal mortality. Hence altogether the U-shaped relationship is often displayed [9], [11].

The estimated nonlinear effect of woman's status is shown in Figure 4. The plot shows slight decreasing effects with increasing status of the woman. The result was surprising. There is a sizeable literature that demonstrates that women with a low status tend to have a weaker control over resources in their households, more restricted access to information and health services, and poorer maternal health [26], [33]. Therefore low women status has a significant negative impact on health outcomes of children. However possible interactions with other covariates such as area of residence is possible and is worthwhile investigating.

**Figure 5:**
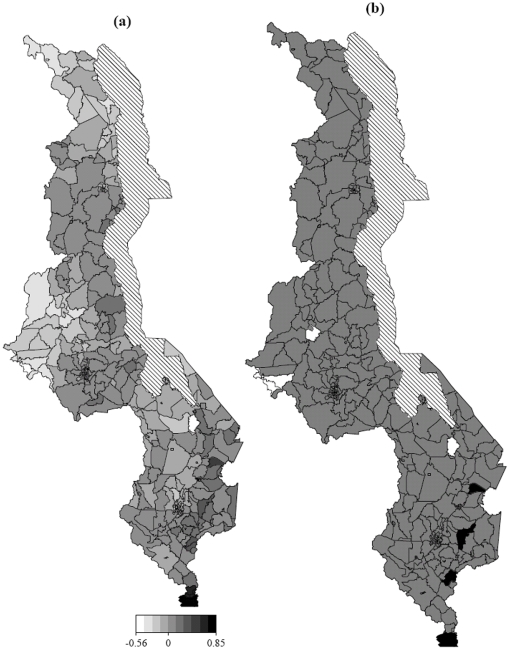
Smoothed geographical effects. (a) Smooth geographical effect (CAR) estimates at district level based on Model 3. (b): Corresponding posterior probabilities at 80% nominal level, white denotes regions with strictly negative credible intervals, black denotes regions with strictly positive credible intervals, and gray depicts regions of nonsignificant effects.

**Figure 6:**
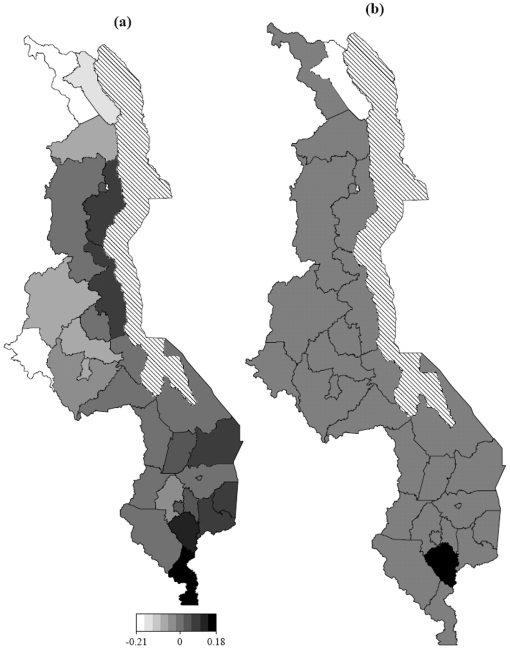
Structured spatial effects. (a) Structured spatial effects, at subdistrict level, of neonatal death (Model M3). Shown are the posterior modes. (b): Corresponding posterior probabilities at 80% nominal level, white denotes regions with strictly negative credible intervals, black denotes regions with strictly positive credible intervals, and gray depicts regions of nonsignificant effects.

**Figure 7:**
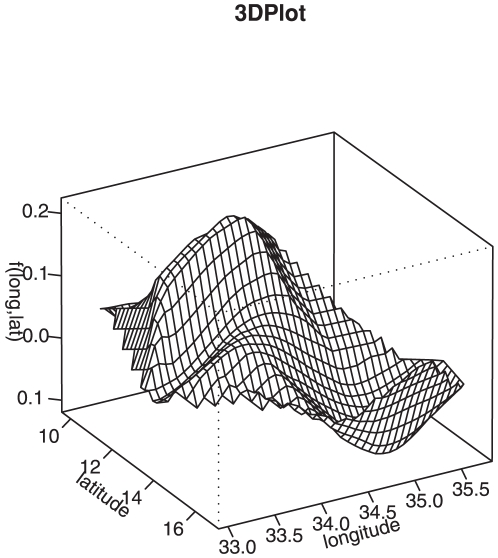
Surface of neonatal disparities. Two dimensional surface of neonatal disparities in Malawi.

Figures 5 and 6 shows the estimated smooth geographical effects at district and sub-district level respectively, after controlling for other covariates. Figure 7 shows the surface interaction plot of the same geographical locations. These represent other risk factors not directly observed, but had an impact on the risk of neonatal mortality risk. These might probably be ecological factors, such as varying deprivation inequalities including severity and depth of poverty, as well as infectious diseases including malaria, HIV/Aids, pneumonia, diarrhoea that directly contribute to the risk of child mortality [1]. These factors often display geographical pattern. As depicted in the map, high risk areas were observed in a number of districts, particularly in Lilongwe, Kasungu and Mchinji in the central region, Mwanza and Chikwawa in the southern region, and Karonga, Rumphi and Chitipa in the northern region of the country. Social deprivation factors might contribute to such high residual spatial effects in our analysis (Figure 6), because they also happened to be the poorest in terms of severity and depth of poverty [34]. This association between social deprivation and the risk of neonatal mortality has been shown in a number of studies. For example in Brazil, similarity between neonatal profile and socioeconomic index have been reported [35]. In many developing countries in sub-Saharan Africa comparable associations have also been observed, see the reviews in References [1], [3], [4], [31]. In general, social deprivation and diseases have consequential effects at attaining quality health, hence reduction in life expectancy [31], [35].

## Discussion

The structured additive regression model combining both spatial random effects and nonparametric offer a flexible approach to quantifying small-scale geographical variability in public health problems. Our objective was to explore small-scale spatial patterns of neonatal mortality. The spatial component was specified through a Markov random fields (MRF) and the two-dimensional P-splines. However, the stationary Gaussian random fields, widely used in geostatistics, is an alternative approach. The models can be represented as mixed models, and can be estimated using empirical Bayesian inference via the penalized likelihood technique. The small-scale geographical disparities in risk of neonatal mortality, thus quantified through the model, may inform evidence-based intervention and policy or further research. The approach we considered also offered a flexible framework which permitted simultaneous modelling of the impact of linear, nonlinear and geographical effects. These model can be extended to more complicated data structures, for example models with space-varying coefficients and of nonlinear interactions. Details and examples of such extensions can be found in Kneib and Fahrmeir [25].

For future research, one may carry out a more explicit comparison between this GLMM approach (where spatial variation not explained by individual-level factors are modelled using spatial random effects) and a main alternative, a multilevel model, whereby the effects of aggregate characteristics of each individual's village and/or district, if available, are considered. Here one may assess if standard multilevel modelling approach accounts for much or all of the spatially structured residual variation compared to the GLMM approach applied in this study. We must add, though, that there is already on-going research in that direction [14], [36], [37].

This study used data from the 2000 DHS. This could be a major limitation considering the data used is almost 10 years old. The landscape of neonatal mortality, as opposed to what we have presented here, may have changed in Malawi, consequently the results may not be sufficiently informative to policy makers. However, our effort should be seen from an attempt to use a novel method in the analysis of health outcomes, and to advance the argument that appropriate models are required to understand and inform on the epidemiology of key health outcomes. Examples of such methods are many in some areas, but lacking in some, for example in neonatal mortality, and the study by Lawn et al. [3], [4] motivates the need to study geographical variation in neonatal mortality. In other words, although most of the fixed factors have been shown in previous studies to influence child mortality in many developing countries, may of such studies do not account for geographical effects. Profiling geographical variations in neonatal mortality is important for scaling-up of targeted interventions.
